# Dialectic narratives, hostile actors, and Earth’s resources in Saskatchewan, Canada

**DOI:** 10.1007/s11625-022-01214-y

**Published:** 2022-09-20

**Authors:** Margot A. Hurlbert, Jane Akpan

**Affiliations:** 1 Canada Research Chair, Energy, Climate Change, and Sustainability Policy, Johnson-Shoyama Graduate School of Public Policy, University of Regina, Regina, SK Canada; 2 Johnson-Shoyama Graduate School of Public Policy, University of Regina, Regina, SK Canada

**Keywords:** Power production, Network of action situations, Actants, Actor network theory, Narrative analysis, Governance, Institutions

## Abstract

This paper uses networks of action situations (NAS) together with actor network theory (ANT) to identify the decisions that were made in Saskatchewan regarding power production and explore what future choices are available in the context of climate change. A theoretical and methodological contribution to NAS literature is made with focus on interconnected human and non-human objects (carbon, hydro, and uranium) or ‘actants’ and the development of discourses supporting or opposing their development. Actants provide the nodes of focus, while discourses explain the development of actants and their links. Identification and explanation of the emergence and recession of actants on the Saskatchewan landscape are analyzed with diagnostics of telecoupled systems, polycentric governance, and flows of faction situations. Focus group and survey data are used to identify future pathways and imaginaries of power production and the actants of carbon, hydro and uranium. Actants of carbon (coal) and hydro are possibly kept alive with carbon capture and storage and import of hydro-electricity from the distant action situation in a neighboring province (if the necessary infrastructure is built). While actants of renewables are strongly emerging, uranium currently mined on the Saskatchewan landscape is receiving strong support by government due to the possibility of small modular reactors (SMRs). This expanded theoretical conception of NAS illustrates distant action situations impact on local narratives and decision-making and dynamics of polycentric governance that are neither top down nor collaborative.

**Supplementary Information:**

The online version contains supplementary material available at 10.1007/s11625-022-01214-y.

## Introduction

This paper answers the question, “what choices were made in Saskatchewan regarding power production in action situations in the past and what future choices are available in the context of climate change?” An empirical case study of networks of action situations (NAS) in Saskatchewan, Canada, is undertaken, mapping out the pathways for utilization of Earth’s resources in Saskatchewan, in the context of power production. The network of action situations both historically, as well as prospectively, are analyzed as actors’ imaginary of future pathways and future resource use, in the context of achieving net zero emissions (given the urgency of climate change). To answer this research question utilizing this method, sub-research questions include the identification of nodes (the actants—which include actors and natural resources) and what are the links (processes of interessement).

Prior to COVID-19, climate change was recognized as the top global risk (World Economic Forum, WEF 2020) and there is little evidence of actors’ concern diminishing even with the pandemic (Evenson et al. 2021). Climate change is a complex problem characterized by multiple stakeholders with conflicting agendas; multi-causal, multi-scalar interconnected attributes; and solutions with unintended consequences that ripple through the socio-ecological system (Olsson and Jerneck 2018). Addressing climate change requires reducing greenhouse gases (GHGs) to zero, and potentially negative emission technologies (Intergovernmental Panel on Climate Change, IPCC 2019). Saskatchewan offers an interesting study as historical focus has been on the actants of carbon (coal and natural gas), hydro, and uranium (nuclear). In 1991, 70% of power production was coal. Although this figure has reduced to only 28%, the future pathway to net zero emissions in Saskatchewan’s power production is not clear and at the current time future options are limited, so that it is increasingly pressing to build an imaginary, or a constructed version of the future (Frose and Mevissen 2020).

This research advances NAS literature (Villamayor-Tomas et al. 2015; Oberlack et al. 2018) by theoretically and methodologically offering an explanation of how and why institutions change (Zikos 2020). The theory of NAS is advanced by considering actants and actor network theory (ANT). ANT extends the analysis of actors by moving beyond the traditional focus of individual personal agency and humans (individually or collectively) affecting social processes to include non-human ‘objects’. By including both the human and non-human actor participants, the considerations of how this combination acts as a durable whole informs the theoretical conception of the study (Latour 2005; Ross and Berkes 2013).

The methods of NAS are also advanced by including actants. NAS have been analyzed with multiple methods including value chain analysis (Villamayor-Tomas et al. 2015), knowledge deficits and networks (Catney et al. 2013), game theory (Kimmich and Villamayor-Tomas 2017), ecology of games (McGinnis 2011), land system science (Oberlack et al. 2018), and discourse networks (Rennkamp et al. 2017). While this research replicates the diagnostic procedure of Oberlack et al. (2018) for analyzing land system science, we expand consideration to that of ‘actant’. In the context of power production and the electricity grid, the inclusion of actants improves the robustness and analysis of choices available to actors in multiple situations; with consideration of actants, future choices of power production can be imagined.

The use of NAS in the power production context fills a gap in literature in determining future pathways for sustainable energy (Devine-Wright and Wiersma 2020; Sovacool et al. 2015; Pidgeon et al. 2014). Social science provides insights into the hopes, concerns, expectations, and resistance underscoring this role (Pellizzone et al. 2017). While there are studies and a rich literature on energy and technology transformations (Geels and Shot 2007; Geels 2010), and Saskatchewan’s socio-technical regime (Hurlbert et al. 2011, 2019, 2020), there is very little research on network structures and NAS in relation to transformation of social–ecological energy systems to sustainable futures.

Commencing with a review of the literature on action situations, actants, and actant networks, this is followed by a methods section that provides a brief description of the Saskatchewan case, its flow-centered system of power production governance, and the methods of this research. The paper proceeds to provide a review of carbon, hydro and uranium and their corresponding dialectic narratives in Saskatchewan, a historical analysis of the flow-centered governance systems to the present, an identification of networks of actions situations, and finally an imaginary of patterns of cooperation, coordination and conflict into the future for achieving sustainability.

## Literature on action situations, actants, and actant networks

Action situations correlate to decision-making spaces, or “social spaces where individuals interact, exchange goods and services, solve problems, dominate one another, or fight (among the many things that individuals do in action situations)” (Ostrom 2011: 11). These spaces are influenced by institutions, or stable, collective patterns of dealing with basic social functions—the rules of the game (Lauer et al. 2006; North 1991) that include formal institutions such as laws or organizations, and informal institutions such as socially shared rules or norms that impact behavior (Hurlbert and Diaz 2013). Key components of action situations include nodes (or vertices) that could include such things as actors, information, biophysical resources, or institutions (Kimmich and Villamayor-Tomás 2017) and links (or strategic interactions) (Therville et al. 2019). Action situations are the building blocks of a governance system, as individual causal mechanisms inform networked and multi sectoral decisions (Baldwin et al. 2019). After a brief discussion of methodological concepts of governance, this section will explain how ANT literature provides insights into analysis of ‘nodes’ and ‘links’.

Action situations and NAS have been used to study the effect of institutions within and across processes in mediating outcomes in many contexts. These include resource governance (water) (Villamayor-Tomas et al. 2015), community energy networks (Catney et al. 2013), irrigation and energy governance (Kimmich and Villamayor-Tomas 2017), lobster fisheries (McGinnis 2011), land systems (Oberlack et al. 2018), and renewable energy support (Rennkamp et al. 2017). Networks of actions situations have been used to address linkages across water, energy and food (Villamayor-Tomas et al. 2015), but have not yet been applied in relation to linkages of power production sources and related resources. This study extends NAS analysis to the resources of water, uranium, carbon, and power production.

**Fig. 1:**
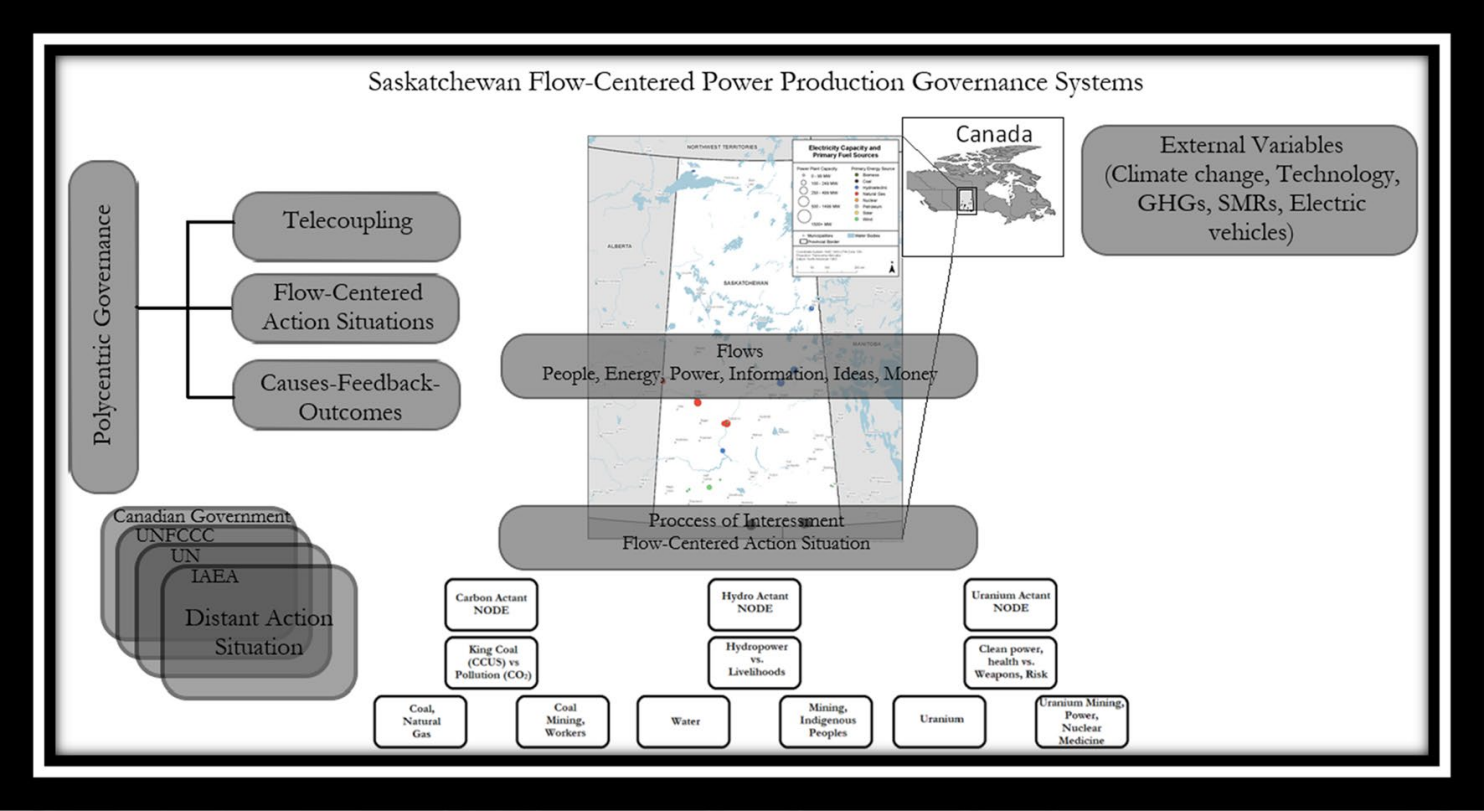
Saskatchewan polycentric flow-centered power production governance systems

**Fig. 2:**
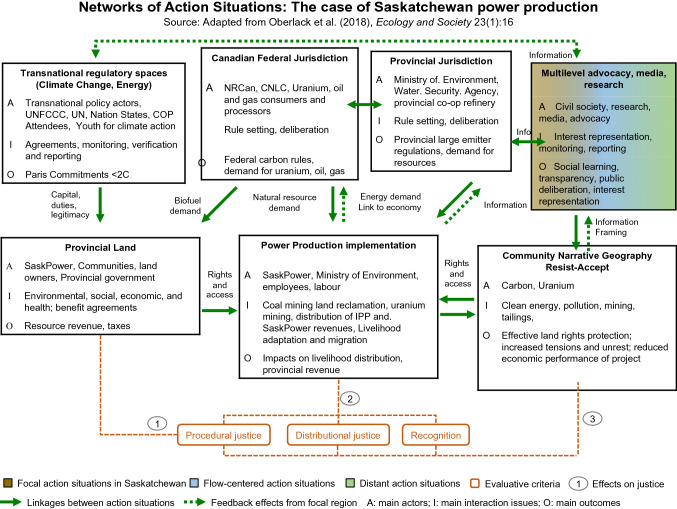
Networks of action situations

Governance “involves formal and informal institutions and it entails the interactions among processes, rules, and traditions that determine how actors in societies make decisions and share power, articulate their interests, exercise responsibility and mediate their differences, and ensure accountability” (Hurlbert and Diaz 2013: 61). Polycentric governance systems (illustrated in Fig. 1) involve multiple arenas of decision-making, that may be in different sectors of the socio-ecological system (water, energy, climate), and are interlinked through processes of conflict, coordination and cooperation (Oberlack et al. 2018). And finally, linking decision-making across institutions and scales can be done using telecoupling. Telecoupling is the interactions over distance of socio-ecological systems whereby distant actors, flows, causes, feedbacks, and outcomes impact local land systems thereby linking process-based analysis of land governance with place-based analysis of land change (ibid). To identify the telecoupled systems and polycentric governance of the NAS for power production, the diagnostic procedure of Oberlack et al. (2018) is followed, as appears in the Methods section, and is illustrated in Fig. 2.

The theoretical contribution to NAS this paper makes is through the ANT literature, the addition of actants (identified as key ‘nodes’) and an expanded conception of networks and their process of assemblage (identified as key processes surrounding ‘links’). An actant is any non-human component (biological, technical or otherwise) exerting influence or agency over the network or relationships constituting society (Risan 1997). To be identified as an actant, the entity must be meaningful within the social system because of the network of relationships it shapes with others, and not the existence of the entity per se (Dwiartama and Rosin 2014). By way of example, Portugal’s naval power for 150 years was due to the agency of actants: ships, and spices (Law 1986). A human food consumer is formed by their relationship with farmers, a supply chain, and the foods eaten. Without these actants, the meaning of the human as food consumer perishes (ibid.) and this is true of non-human actants as well. In the energy socio-ecological system humans are power consumers with a unique relationship with the contextual Saskatchewan actants of power production resources [water, coal (carbon) and uranium]. These three actants are identified in Fig. 1 in the context of Saskatchewan.

The addition of the concept of actant also brings with it an expansion of the concept of networks. Actor network theory (ANT) focuses on studying not only people or actors in examining society, but also the relations between people and the material objects or ‘actants’ surrounding them (Latour 2005; Latour and Johnson 1988). “The dualism between humans and objects is transgressed: everything—actors, machines, ideas, etc.—is treated symmetrically and explored as interactional effects” (Nyborg and Ropke 2013, p. 167). Interactional, or network effects refers to the relations of entities with other entities; entities have no inherent qualities, attributes or agency on their own (Wong 2016). ANT extends the participants from actors in the social [beyond the traditional focus of individual personal agency and humans (individually or collectively) affecting social processes] to include objects, such that the human and non-human actor participants act as a durable whole (Latour 2005; Ross and Berkes 2013). Decision-making is thus informed and potentially constrained by the actant. Thus, actants or material objects reflect a structure influencing individual agency and decision-making in multiple situations, and expands the analysis in relation to ‘nodes’ by identifying key coupling of actors and biophysical resources.

The addition of actant and ANT to NAS arguably deepens understanding of agency and groups. Further it assists with delineating boundaries of action situations, offering explanation of the interplay of actors and actants in telecoupling. The conflicts and narratives entailed in the identification and analysis of actors and actants adds explanation to boundaries of action situations including patterns of cooperation, coordination and conflict, offering another method of operationalizing telecoupled systems: that of narrative analysis.

ANT proposes that human actors and non-human actants develop social ordering or structure, and the rigidity or fluidity of the structure of these networks depends on the way the actants continuously form networks among each other (Murdoch 1998). These networks are ordered relationally, as human and technical components form a unified whole through complex, dynamic, constant coupling, and continuous flows of otherwise fragmentary objects (Muller 2015). The idea of flow-centered action situations is illustrated in Fig. 1.

This process of ordering or creating a network ‘assemblage’ is also called a process of translation or ‘interessement’, whereby the network assemblage gains credibility or solidifies. Interessement begins with problematization, or the framing of an assemblage as vital to addressing a pressing problem (Latour 2005). Actors must regard an actant as necessary for their interests and a particular storyline and scenario evolves (Callon 1986). This problematization creates roles and identities for each actor/actant in the network, thus giving a degree of stability and relevance to the actant (Mahring et al. 2004). It is then through a process of interessement that actors and their support structures enroll others thereby expanding and strengthening their networks through a subtle practice of indoctrination and enlistment (Ambrose et al. 2016; Boelens 2010; Latour 1987). The actor network process of assemblage deepens analysis of actor network situations and ‘links’ or strategic interactions.

Through analysis of NAS through identification of nodes (actants) and links (processes of interessement) that are crystalized through flow-centered action situations, this paper answers the question, “what choices were made in Saskatchewan regarding power production in action situations in the past and what future choices are available in the context of climate change?”.

## Methods

Quantitative and qualitative methods inform this analysis of network of action situations in the Saskatchewan case offering in-depth, multiple, unique, and complex perspectives of a real life socio-ecological system (Thomas 2011). Historical analysis of the resource sector of Saskatchewan is based on secondary data and previous in-depth case studies of Saskatchewan (Hurlbert et al. 2011, 2019, 2020) and an analysis of discourse. Discourse includes ideas, narratives, and framing of values (Schmidt 2010).

A mixed methods study was employed, using online survey and focused group discussion to understand explanatory narratives and perspectives within participants, and offer additional insight into the research questions (Cresswell and Creswell 2018).

The objective of the survey was to provide a measurable result of the actors’ thoughts in the context of power production sources (actants) in the future. The survey comprises adult residents of Saskatchewan, randomly selected based on published Saskatchewan telephone numbers conducted in July and August 2020. In this phase 400 respondents were contacted and 136 (34%) agreed to fill in the survey. The survey comprised Likert-scaled questions duly analyzed in Online Appendix. The survey complements results from focus group discussions by providing some level of statistical information about the perceptions of actors and possible power production sources (actants) in the future, anonymously.

Analysis of the focus groups revolved around coding themes using narrative analysis. Narratives, or recurring stories in political debates, are important in analysis of energy systems (Trencher et al. 2019). The identification of narratives ascribed to the durable whole of the actant (human and non-human) (Latour 2005; Ross and Berkes 2013) explains decisions surrounding the actants and specifically the dominance of some actants over others. The process of translation and interessement of actors and actant networks was analyzed through discourse or institutionalized structures of meaning that channeled political thought as well as action (Connolly 1983), including technical and scientific arguments, with more generally accessible narratives or compelling stories (Schmidt 2002).

Participants were selected as detailed in the Online Appendix to represent Saskatchewan residents geographically by selecting ten communities dispersed throughout Saskatchewan. Transcripts of focus groups were coded utilizing the codes in Online Appendix. Codes were determined based on the in-depth review of secondary sources relating to Saskatchewan’s resource and power production sector and the identified actants (coal, water and uranium) in relation to power production. Coding was carried out in order to determine discourse—the system of statements constructing an object (or actant) (Cheek 2004).

Focus group data included a qualitative analysis of focus group discussions. Focus groups provided a deep understanding of participant’s ‘imaginary’ of what achieving net zero emissions might look like in the future by facilitated exchange of participants’ opinions. Combining focus groups with survey offers researchers the ability to hear in-depth views and voices of participants and conduct a qualitative narrative analysis, while also analyzing conclusions quantitatively (Morgan 1996). The limitation of this research is that it is not a representative sample of the Saskatchewan population.

## Results

This section provides a detailed analysis of the decisions that were made in Saskatchewan regarding power production and also highlights the future choices that are available in the context of climate change, thereby addressing the research question. First, the actors and actants are identified together with historic narratives and network assemblages surrounding the actants of carbon, water and uranium within the province of Saskatchewan. The polycentric and flow-centered governance systems that links networks of actions situations and pertains to the carbon, hydro, and uranium (nuclear) actants and energy system in Saskatchewan, Canada to the present are analyzed. This sets the stage for analysis of the existent NAS and deliberative fora. Survey and focus group data provide the basis for identification of potential patterns of cooperation, coordination and conflict, or imaginaries for future pathways.

### The polycentric power production governance system and carbon, hydro, and uranium actants

Achieving net zero carbon emissions required to maintain climate warming well below 2 °C will be significantly challenging in Saskatchewan given its long cold, dark winters where temperatures often reach minus 40 °C and there is a fossil fuel dependence (Peszko et al. 2020). The research question of how Saskatchewan will achieve net zero emissions in power production appears and is diagnosed in Table 1.

**Table 1:** Diagnostic of telecoupled system and polycentric governance for integrated analysis using networks of action situations for Saskatchewan power production adapted from Oberlack et al. (2018)

Step	Question
1	*Issues and research question*: Achieving net zero carbon emissions in power production in Saskatchewan—a social–ecological energy system (with specific focus on carbon (coal and natural gas), hydro, and uranium (nuclear), Canada What choices were made in Saskatchewan regarding power production in action situations in the past and what future choices are available in the context of climate change?”
*Land system in focal region*	
2	*Nodes (Actants, Actors) and Links (Agency, Interessement)*: Actants—carbon, water, uranium; Actors—SaskPower, Sk Government, Federal government, Mining companies, communities, Indigenous people, people; Agency—SaskPower monopoly, Independent power producers market entry, actant narratives, interessement (translation and narrative building) outlined below
3	*Ecological processes* in respect of sustainability challenge identified in step 1: impacts of climate change, reduction in hydro resource, downstream hydroelectric implications, depleting oil reserves, cold winters with reduced solar resource
4	*Institutions* regulating interactions in the focal Saskatchewan Environmental Society (SES): Dominant narratives, Paris commitments (UNFCCC), carbon price, power production, transmission and distribution planning, technology innovation policy, Indigenous livelihoods, Indigenous and treaty rights
*Flows and flow-centered governance systems*	
5	*Flows* link focal region with distant regions. How flow-centered governance systems shape flows: climate change impacts (including inter/intragenerational), GHGs, International GHG commitments, constitutional division of powers, financial/trade of oil/gas, uranium, hydro
*Distant regions*	
6	How ecological, socioeconomic and institutional factors shape interactions and outcomes in distant action situations including connecting flows? Infrastructure and technology, financial/debt burdens, Northern Manitoba hydro development, technology innovation
*Network of action situations (focal, distant and flow-centered)*	
7	Focal, distant and flow-centered action situations, narratives of actants, and processes of interessement, affect power production sources and their governance including interactions, linkages and outcomes

Saskatchewan is a province in Canada located in the center of the Canadian Prairies. Distant action situations including decisions occurring in the United Nations (UN) with the establishment of the Sustainable Development Goals (SDGs), at the United Nations Framework Convention on Climate Change (UNFCCC) and at the International Atomic Energy Association (IAEA) in Vienna have implications for the water, energy and food production systems in Saskatchewan. Paris commitments impact the choice of power production sources in Saskatchewan and has impacts on resources (oil and gas production, uranium mining) and the flows of goods and money through national and international trade. Action situations, polycentric governance, and telecouplings in the Saskatchewan, Canada, energy case appears in Fig. 1.

The actants of carbon (coal), hydro, and uranium are selected based on a review of secondary sources surrounding Saskatchewan’s power production system and identification of Saskatchewan’s prominent energy resources. Dependence on coal is due to vast lignite coal, oil and gas located in the southern part of the province (SaskPower 2010). The Saskatchewan north is home of the world’s second largest uranium mining industry although Saskatchewan has no nuclear power generation (The Canadian Encyclopedia, TCE 2020). In Saskatchewan’s north, 42.8% of the population identify as Indigenous (compared to 16.3% of the Saskatchewan population and 4.9% of Canadians) (Statistics Canada 2018). 12% of the province is covered by lakes and rivers, but there are no significant hydroelectric resources partly due to its relatively flat topography (Widdis 2006). Reliability of power production is challenged by Saskatchewan’s relatively sparse population of one million people and its land mass of 588,239 km^2^ (TCE 2020). Combinations of renewables, carbon capture and sequestration (CCS) technology (Koelbl et al. 2014), nuclear, and purchasing more hydro-electricity from the neighboring province of Manitoba (together with building massive transmission infrastructure) are Saskatchewan’s future options (Djuric 2019).

The provincial utility is subject to increasing regulatory requirements to shut down coal without CCS by 2030, and not build any new natural gas (eventually facing their closure as well) from telecoupling and the distant action situations and flows identified in Fig. 1 surrounding climate change, (CER 2020). For these reasons, the actants of coal (carbon), water (hydro), and uranium (nuclear) are particularly germane and are the ‘nodes’ of analysis of NAS in relation to Saskatchewan power production.

### Dialectic narrative hostilities of carbon, water and uranium actants and their network assemblages

The predominant actors and their network in Saskatchewan’s energy and power production system appear in Fig. 2. Historically, coal emerged as an asset, but its derivative, CO_2_ is both classified as toxic and a pollutant, as well as a commodity, or asset at the same time (International Energy Agency, IEA 2010). Similarly, dialectic hostile narratives exist in relation to hydro and uranium. Each will be discussed in turn.

SaskPower represents a century of mergers of municipal utilities, acquisitions of power plants, and expansion of transmission and distribution joining a sparse rural population (White 1976) through processes of interessement based on cooperation. Up until the 1970s, this provincial utility was built based on the model of centralized coal power production (White 1976; Rediger 2004). A dialectical and conflictual narrative of coal as ‘dirty’ began to emerge, challenging its assent to ‘king’ due to environmental and air quality concerns as early as the 1970s (Saskatchewan Mineral Resources, SMR 1981) and the release of CO_2_ (SaskPower 1991, 1995b). The dialectic narrative of coal and its associated CO_2_ portrayed it as a poison, waste, and as ‘toxic’ to the environment as designed by the *Canadian Environmental Protection Act* (CEPA) 1999 (s. 64(a)).^1^

CO_2_ is recognized as a commodity and asset, especially when deployed in Enhanced Oil Recovery (EOR) or other industrial operations (as it has been in Saskatchewan for the last three decades) and carbon capture utilization and storage (CCUS) (Hurlbert et al. 2020). Here, CO_2_ becomes an input in an industrial process which transforms it from a toxic waste into a commodity. In another context soil is an important sink for CO_2_. Soil carbon sequestration occurs through soil conservation practices that reduce soil erosion and increase the soil organic material content of soils. Converting marginal crop lands to perennial native systems or rangeland, practicing no-till or conservation-till farming, reducing the frequency of summer fallow in crop rotation and incorporating manure into soil (Miller 2013).

Saskatchewan’s rich water resources have provided livelihoods to Indigenous people from time immemorial and continue to be foundational to Indigenous rights of hunting, trapping and fishing. Hydro-electric power generation commenced as early as 1930 in Saskatchewan to support mining (Marshall 1931). However clean hydro-power has trade-offs with fresh-water biodiversity regionally (Jumani et al. 2017) and food security locally (with loss of fishing) (Turney and Fthenakis 2011) reducing downstream ecosystem services (Zarfl et al. 2015).

Since the 1970s the narrative surrounding the actant of hydro has been conflictual. Two boards of inquiry were established in 1977 to review SaskPower’s plans to build two new dams. While one proceeded subject to conditions (eventually completed in 1985), the other did not (Rediger 2004; Abouguendia et al. 1980). The rejection came after a Federated Saskatchewan Indian (FSI) Study concluded degradation of the environment caused by the dam would reduce the productivity of hunting, trapping and fishing activities and ‘contribute to the destruction’ of the Crees’ way of life (Saskatchewan Indian 1977, p.1). This dialectic narrative opposing hydro-electricity that harms socio-ecological downstream livelihoods continues to today (Warick 2020).

The world’s second largest producer of uranium is northern Saskatchewan with the world’s largest deposits of high-grade uranium. 85% of production is exported, while 15% is used in nuclear power production in Ontario and New Brunswick (Government of Canada, GOC 2021). These nuclear plants, together with cyclotrons and linear accelerators, support an interessement and a cooperative narrative surrounding the actant of nuclear, providing medical isotopes used for cancer radiation treatments, sterilizing medical devices, treatment of food and consumer products, and medical diagnostic procedures (GOC 2021).

Opposing the resource narrative of uranium is the dialectic narrative surrounding nuclear power which includes risk, disarmament protests, nuclear accident, and storage of nuclear waste (Harding 2007). These two narratives clashed in Saskatchewan most recently in 2009 when the Government of Saskatchewan released a commissioned report proposing a large nuclear plant as well as many other initiatives advancing the uranium industry in Saskatchewan (Uranium Development Partnership, UDP 2009). Public discussions ensued both through a Legislative Committee investigating how to best meet Saskatchewan’s future energy needs and a public consultation throughout communities in Saskatchewan in relation to the commissioned report. While a large nuclear power facility did not proceed, smaller initiatives did (Hurlbert 2014). At present, Saskatchewan is one of four provinces who have signed a memorandum of understanding to coordinate in exploration of small modular nuclear reactors (SMRs) and demonstration projects are advancing with potential availability in 2026 (Djuric 2019).

### Flow-centered governance systems and actants

This evolution of Saskatchewan’s landscape of power production and the actants of carbon (coal and natural gas), hydro, and uranium (nuclear) span just over a century. While rich lignite coal deposits have long been mined for heating, they transitioned to power production in the mid-twentieth century. Nickel and gold mining in the north also transitioned to include uranium at this time. Hydro-electric power production was originally developed to support this mining and since has expanded into SaskPower’s power production system.

As depicted in Fig. 1 (Saskatchewan’s polycentric and flow-centered governance systems), the external variables which include agreements, protocols, and treaties impacting water, coal, and uranium have evolved since the mid-twentieth century. In Fig. 2 these variables are specifically listed to include transnational, Canadian (federal), Provincial, and multilevel actors. Canada’s constitutional division of powers makes for an interesting flow. While the federal government has jurisdiction over nuclear and the Canadian Nuclear Safety Commission (GOC 2021) and inter-jurisdictional issues (flows of water, trade, toxic pollution), provinces have jurisdiction over natural resources within their boundaries including property and civil rights, power production and distribution within the province, local works and provincial corporations (Hurlbert and Diaz 2013; Hogg 1985). Natural resource demand is provincial, but power, uranium, and oil and gas are exported from the province and have supported the interessement of the actants of carbon and uranium (nuclear).

In the late 1940s and 1950s Saskatchewan developed a nuclear medicine program having one of Canada’s two Cobalt-60 teletherapy devices for treating cancer in 1951. The first patient was treated that year. That patient survived the next 37 years. This technology was exported; by 1981 there were 2,200 such units being used in the free world. Eldorado Mining & Company (a predecessor of Cameco Corporation) was the resource provider (Fedoruk 1989). Eldorado and later Cameco continue to export uranium to Eastern Canada where it is processed and used in nuclear power generation in Ontario and New Brunswick. Approximately 70% of Ontario’s power production is generated with nuclear power.

In 1997, the federal government signed the Kyoto Protocol committing to the reduction of GHGs; foreshadowing this, SaskPower decided to abandon building the second coal-fired power generation station at Shand in Estevan in 1993 (Hurlbert et al. 2010). It was not until later that federal regulations were passed restricting the amount of emissions from coal-fired power generation (Environment and Climate Change Canada, ECCC 2012; Canadian Environmental Protection Act, CEPA 1999). The continued tightening of these regulations has resulted in the decommissioning of two further coal generating units in 2013 and 2015 (SaskPower 2016) and Canada joined the global alliance to phase out coal by 2030 (UNFCCC News 2017), illustrating the telecoupled and polycentric nature of distant action situations influence on the power production landscape in Saskatchewan.

Reversing a century of interessement and the building of a monopolized SaskPower power production and distribution company (wherein multiple small municipal and private generation and distribution companies were amalgamated from 1927–1991) (Hurlbert et al. 2020), SaskPower’s coal divestiture resulted in the entry of new actors by way of Independent Power Producers (IPPs). Many different options and opinions about the replacement of coal have been discussed in Saskatchewan, however no one-technology has emerged (SaskPower 1991; Saskatchewan Energy Conservation and Development Authority, SECDA 2003; SaskPower 1995b). After 1994, SaskPower waived its exclusive franchise and contracted for supply first from the Meridian power project owned by TransAlta and Husky and then wind energy from Sunbridge (SaskPower Sunbridge Wind project). As a result, new actors have emerged building gas and wind power plants (SECDA 2003).

Since the displacement of coal, numerous technological niches have emerged including co-generation, natural gas, wind, and biomass. A gradual replacement of coal-fired generation by natural gas and wind generation has occurred. Dolter and Rivers (2018) conclude that a combination of new transmission and renewables (wind, solar and hydropower) is optimized at prices of $80/tonne CO_2_e. In the future, they predict that as the price of carbon increases, eventually it will surpass a price point that will make natural gas uneconomic (ibid.).

The flows of finance have been important as restrictions on SaskPower and the province’s access to finance have resulted in further reliance on IPPs, and some downsizing, cost-cutting, and performance improvement at SaskPower (SaskPower 1995a). The flow of energy through transmission lines connecting SaskPower to the United States of America and desire to retain profitable electricity wheeling services, which provide for greater grid reliability, resulted in deregulation of Saskatchewan’s wholesale electricity regime (Saskatchewan Energy and Mines 1994).

Southern Saskatchewan is highly engaged and dependent on the oil and gas industry, continuing to sustain the actant of carbon.^2^ A 110 MW CCS plant was retrofitted into the SaskPower’s Boundary Dam plant in Estevan, Saskatchewan in 2014, and in June of 2015 a carbon capture test facility was launched at SaskPower’s Shand Power Station (Fleece 2015; Canadian Electricity Association 2017). The narrative that imagined and advanced this network exemplifies interessement. First, international support for CCS (IEA 2010; Choptiany et al. 2014) was buttressed with arguments and public representations supporting CCS due to Saskatchewan’s “300-year supply of coal” that the government was interested in using to continue to provide base-load power (Legislative Assembly of Saskatchewan, LAS 2013). CCS was defended as it protected coal mining jobs and jobs at the Boundary Dam coal power generation unit (LAS 2015a). International actors including BHP Billiton and contracts with neighboring States created alliances (BHP Billiton 2016; Graham 2017). Even the Saskatchewan Environmental Society supported the CCS project stating that unless coal-fired units were equipped with CCS, they should be de-commissioned (Halliday 2013). In December of 2015, a few weeks before attendance at the United Nations Climate Change Conference in Paris, Saskatchewan Premier Wall announced SaskPower’s plan to rely on 50% renewable energy for electricity supply by 2030 by ramping up of solar, wind, hydro, and geothermal (LAS 2015b; SaskPower 2021).

### Networks of action situations (deliberative fora)

Internationally, the UNFCCC has impacted the NAS at the federal and provincial levels. As early as the 1970s, damaging CO_2_ in coal prohibited any further expansion. At the federal level, the government of Canada is embarking on achieving net zero carbon emissions by 2050. The Canadian Net-Zero Emissions Accountability Act, C-12 (2020) was introduced to Parliament in November 2019 to make the goal legally binding by 2050.

**Fig. 3:**
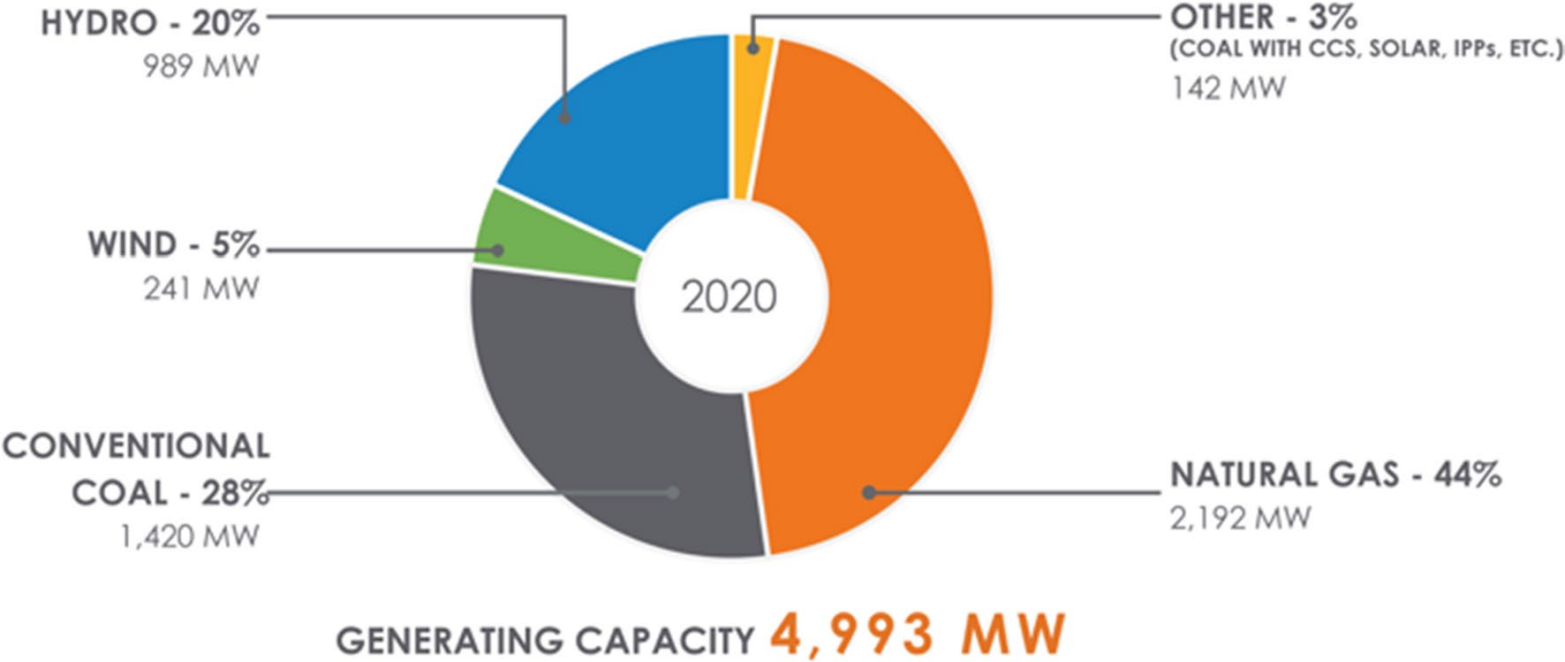
Saskatchewan’s 2020 power mix

Saskatchewan’s landscape has changed and is still changing. After the fledgling CCS Boundary Dam (110 MW) project, no further CCS has been developed in Saskatchewan. The only possible remaining coal plant (Shand) is only 276 MW, a fraction of Saskatchewan’s power production as depicted in Fig. 3.

Significant wind expansion is occurring throughout the province and there is potential for replacing coal generation in Estevan with industrial scale solar and SMRs. Although carbon is in decline, there is argument that Saskatchewan is missing opportunity. Not only do climate change scenarios call for CCS technology but in order to maintain global warming below two degrees Celsius, scenarios rely on negative emission technologies, and Bioenergy CCS is one of these (IPCC 2019). With Saskatchewan’s vast forest resources, the opportunity to convert power production from coal to bioenergy and inject bioenergy CO_2_ into existing deep underground storage may be a missed opportunity. It is unclear why this narrative has not resonated in Saskatchewan, but one hypothesis is the predominance of the oil and gas industry (TCE 2020).

New provisions increasing the price of carbon, however, may make bioenergy CCS technology viable. The increase of the carbon price to $170.00 by 2030 makes this innovative technology viable. The Saskatchewan government intends to address climate change through adaptation and innovation including new technologies such as SMRs and CCS (which would need to be deployed on natural gas) (GS 2016; GS 2017c).

SaskPower is still governed by cabinet decision items made by the Saskatchewan government. Future decisions will determine whether climate commitments are met as some coal, gas and wind installations approach end of life capacity and 1,820 MW of dependable capacity must be replaced due to retirements and expiry of power purchase agreements (SaskPower 2021). The emergence of the electric vehicle further problematizes renewables. In a jurisdiction such as Saskatchewan, the substitution of electric cars for gasoline cars (potentially natural gas powered) is of arguable utility. However, if Saskatchewan’s transportation sector becomes electric, the increase in demand in the electricity sector, in a manner adhering to commitments made in Paris, increases the required power generation of SaskPower’ electrical grid (Navius Research Incorporated, NRI 2016). Planned wind and solar installations may not be enough.^3^

### Future pathways: imaginaries of the actants of coal, hydro and uranium

The main actants (Fig. 1) and actors (Fig. 2) remain significant in Saskatchewan. However, the climate change crisis, together with increasingly restrictive Canadian (and potentially UNFCCC commitments) establish a landscape of change. As hypothesized by Zikos (2020), emerging conflicts surrounding Saskatchewan’s actants can be identified through the interdependency of characteristics of the actants (as natural resources) and their users, in the context of climate change.

A net zero carbon future by 2050 establishes a unique distant action situation characterized by time. This narrative arguably disrupts the dialectic outlined previously in relation to the good and the bad of carbon, hydro, and uranium. Both the crisis situation surrounding climate change, and the relatively short timeline for achieving net zero carbon emissions also changes polycentric governance by decentering the deliberative fora of NAS. Traditional decision-making fora are destabilized and expanded because of high-risk considerations of climate change impacts and energy justice considerations in the fossil fuel economy transition. A new political dimension is added with the loss of jobs in southern Saskatchewan’s traditional fossil fuel economy and addition of jobs in the neighboring province supplying hydro-electricity to replace coal. As power production previously has been dominated by decision-making by the provincial government and SaskPower, the opportunity emerges for new provincial action situations and narratives around power production implementation, with regional land and community implications.

Using survey and focus group data the emerging Saskatchewan narrative is provided followed by an analysis of its potential translation based on Saskatchewan’s historic dialectic narratives of carbon, hydro and uranium. From this data, we construct a ‘future imaginary’ of power production in Saskatchewan, supported by the framing of the assemblage of power production in Saskatchewan, the evidence supporting it, the alliance of actors and their links, or process of interessement (Ambrose et al. 2016; Boelens 2010).

#### Carbon (natural gas with CCS) replaced by renewables

**Fig. 4:**
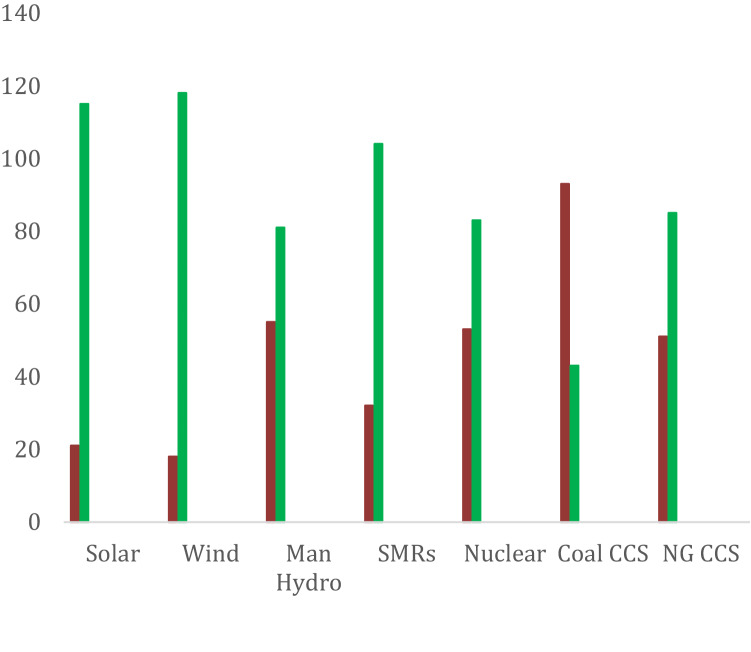
Level of support (green) and lack of support (red) for power production sources

Carbon, or CO_2_, continues to be dominated by narratives of conflict. Irrespective of the emergence of the necessary interessement that included the enhanced oil recovery process that united the actors SaskPower, and the oil and gas industry with the climate change narrative, this alignment with the carbon actant is not enduring. Figure 4 shows the majority of respondents are not in favor of coal with CCS. The interessement that had emerged in 2014 surrounding CCS (and the first post-combustion coal CCUS plant) has been eroded by lack of trust in the oil, gas and coal industry. Many focus group participants believed coal with CCS advanced the fossil fuel industry. One stated it was a “waste of effort. The world is going elsewhere and not looking back.”

However, there is a potential that CCS and CCUS may emerge as an asset. As Canada’s carbon tax increases to $170.00 per tonne by 2030 (ECCC 2020), natural gas and CCS may become feasible and a bridge to net zero by 2050. This option was acceptable to a majority of respondents (Fig. 4). Processes of cooperation exist as international and federal actors continue to support CCS with leadership forums, tax credits, and subsidies. The International Energy Agency identifies CCS as the only technology that can mitigate GHG emissions from large-scale fossil fuel use and could account for 20% of GHG reductions needed by 2050 (IEA 2010).

Regardless of the place of CCS and CCUS [with natural gas (the new fossil fuel source)], the narrative necessary for CO_2_ to be viewed as a resource is fragile, while those for renewable energy are strong. Renewables which include wind and solar are necessary and the province plans to increase renewable energy sources up to 50% of SaskPower’s generation capacity (SaskPower 2021) as illustrated in Fig. 3.

#### Hydro, replaced with import from Manitoba hydro, but concerns remain

There were slightly more people in favor of imports from Manitoba Hydro than against, but only marginally so. Opposition centers around loss of jobs and energy justice security (an emerging narrative). The pre-existing conflictual narrative still exists; there were many people that discussed the negative impacts of hydro-electricity on Indigenous livelihoods and the environment:

And that affects mostly First Nations rights, because it's there for reserves on that area that are affected ...

And as important as hydroelectricity is, and as clean as it can be, there are still impacts along the way. You use hydroelectricity, there’s a lot of concrete gets poured. There’s usually flooding that’s involved. There’s mercury released into lakes for any fisheries that were once pursued.

Regardless of this conflict, SaskPower is moving ahead with new contracts to import hydro-electricity from Manitoba, in small increments totaling 315 MW (albeit a fraction of Saskatchewan’s 5000 MW requirements) in the next few years (Manitoba Hydro 2020).

#### Uranium (nuclear)

Nuclear energy is not clearly in Saskatchewan’s future. The majority of the survey respondents were very positive or very ‘interested’ in respect to SMRs and advances made in nuclear technology. However, education and information were identified as key future requirements, and much was needed. One participant linked Saskatchewan’s uranium mining history with the possibility of SMRs as expressed below:

Coming back to the act in order to actually do any of this. I mean, my husband, by the way, worked in the nuclear energy industry as well. So, way back then at Key Lake, and something he did all the process designs, things like that. And I think coming back to it, it’s not just the cost that the public will be concerned about on the basis of which we will say, let’s go ahead with that. It is to understand that now, relatively speaking, in a fairly safe industry, letting people just have this idea that is the most harmful industry at all. I mean, that I think I would stress that education is absolutely crucial of the advances that have been made since the beginning of when some of these other nuclear reactors were built, and have caused problems. But nowadays, the technology is not at all comparable to that. So, we need to talk about that and educate the public about that.

Institutional developments to support this interessement are occurring with new government ministries, government organized SMR action plans, inter-provincial agreements for SMR advancement and SMR demonstration projects (World Nuclear News, WNN 2020; Natural Resources Canada, NRCAN 2020). However, optimism is tempered by a minority of people concerned or opposing nuclear energy and unsure about SMRs (Saskatchewan Environmental Society, SES 2021). Survey respondents’ questions included, “what exactly SMRs are, how they operate, and how they differ or are similar to Canada’s current nuclear power plants.” There is still significant uncertainty surrounding SMRs, nuclear technology and uranium in the province.

The Saskatchewan government is clearly on the path of uranium and SMR development. Saskatchewan’s Growth Plan (2020) projects Saskatchewan uranium sales will grow to 2$ Billion annually by 2030 and links this to the expansion of SMRs, GS (2020) including SMRs for use in Saskatchewan (Bramadat-Willcock 2020). Additionally, Cameco, one of Saskatchewan’s uranium producers has entered into a partnership to form a research hub exploring innovations and SMRs as well as an interest in the necessary uranium enrichment technology supporting SMRs (Cameco 2021). While some Indigenous people are skeptical of the need for SMRs (Bramadat-Willcock 2020), Indigenous communities adjacent to uranium mining are generally supportive (Deranger 2010).

## Discussion

This research advances NAS literature (Villamayor-Tomas et al. 2015; Oberlack et al. 2018) theoretically and methodologically, offering an explanation of how and why institutions change (in this study the institutions of power production in Saskatchewan) (Zikos 2020). This study offers two innovations: First, we focus on actors as ‘nodes’, and expand the conception to ‘actants’ and include salient combinations of actors and biophysical resources. By employing the ‘actant’ coupling, vertices connecting sectors through telecoupling and polycentric multi-level governance can be identified. Secondly, we studied ‘links’ of action situations through the processes of interessement, or the building of a narrative of support or contestation for an actant. By studying telecouplings as well as the interconnections between actants, we highlight strategic interactions and the emergence, or reduction of actants on the Saskatchewan landscape. Dialectic, conflicting narratives and cooperative coordinating narratives (building interessement) methodologically explained past decision-making. We used focus group and survey data to identify potential imaginary futures.

In this study climate change, and specifically Canadian federal government policies, and international UN and UNFCCC norms and rules telecoupled, or interacted over distance and scale with the Saskatchewan power production regime, building conflict over carbon as an actant, building support for renewables, and offering the potential of CCUS. Thus no one clear government (federal or provincial), with various jurisdictions over the sectors studied (see “Dialectic narrative hostilities of carbon, water and uranium actants and their network assemblages”) could be identified as invoking top down, or collaborative decision-making measures. This diverges from findings such as Zikos (2020) which identified these types of processes in relation to resource conflicts. An analysis of actants and interessement in this research has demonstrated the nature of polycentric governance systems. Specifically identified in this case study are influential Conference of Parties (COP) decisions at the UNFCCC level, the Canadian government level, and the provincial level, as well as the land (geographical) level (in relation to resource endowment and its development). Historically in Saskatchewan, processes of conflict, coordination and cooperation have occurred across these distant action situations (Oberlack et al. 2018) and deliberative fora.

**Fig. 5:**
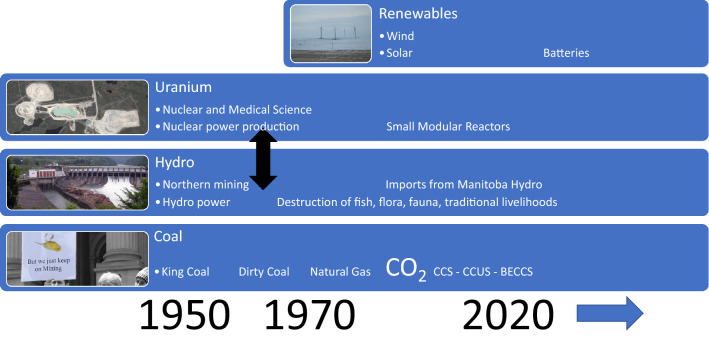
Saskatchewan actants, decisions over time, and future imaginaries

As illustrated in Fig. 5, in the mid-1970s processes of conflict in relation to coal and climate change allowed ‘King Coal’ as a narrative to be replaced with ‘Dirty Coal’. However, strong local coal narratives, together with distant action situations including international and federal calls for coal phase out, achieved the first post-combustion power production CCS plant. A process of interessement advanced this first CCS plant in Saskatchewan, and it is possible that this process could occur again, but the narrative of ‘Dirty Coal’ predominates based on survey and focus group data. There is some support for natural gas with CCUS potentially ensuring the survival of the carbon actant.

The influence of local context on institutions and within the polycentric governance system is surprising. Rich northern uranium resources launched nuclear medicine and science in Saskatchewan with export of cancer treating technologies, but local nuclear power production has not yet developed because of links of conflicting narratives within the actant of uranium (nuclear). Local context is also important in relation to perceptions of hydro. The provinces’ hydro resources were initially synergistically developed with northern mining, but by the 1970s regarded as environmentally harmful and livelihood destroying, damaging fish, and other flora and fauna. Already the persistence of this link is changing as the province imports hydro from Manitoba, albeit in a small quantity.

But strong renewable energy support exists throughout the province. Local and international interessement has firmly established this actant. Just days before the 2015 Paris UNFCCC the provincial government announced a 50% renewable energy target by 2030. Unsurprisingly, strong actants of wind and solar have emerged. No significant local opposition has yet emerged (an obstacle in other locals, as identified by Devine-Wright and Wiersma (2020)).

Survey and focus group data provide the basis for future imaginaries and actants. The data also provide the basis for identification of potential patterns of interessement, or cooperation, coordination and conflict for future pathways involving the actants of CO_2_, uranium, and hydro. The actants of carbon, uranium, and hydro from Manitoba are still possibilities in Saskatchewan’s landscape. Although not yet pervasive, renewables (approximately 5% of power production) may emerge as a future actant because of the strong interessement that has been built supporting it, together with batteries for energy storage. Recognizing international discourses, there is a potential that CO_2_ will emerge as an asset and CCS and CCUS will expand (being deployed in relation to natural gas). The narrative around SMRs is equally strong as that for importing hydro from Manitoba, but the former is strongly supported by the national and provincial governments and the mining industry. The pervasiveness of future distant action situations supporting the actant of uranium, versus hydro from Manitoba, remains an open question. It may be that the actant of uranium interacts with national actions situations to expand its presence in the province.

## Conclusion

This paper documents the choices made in Saskatchewan regarding power production in action situations in the past, and possibilities for the future. Abundance of coal and actors’ support of its development gave rise to the actant of ‘King coal’; however, its interessement has eroded due to conflict with climate change, allowing renewables (solar and wind) to emerge. Saskatchewan’s actant of uranium has supported nuclear power production elsewhere and interessement is growing for it in the province. However, what looks like a future of renewables, and possibly nuclear, could still be displaced by the actants of coal and hydro. Future development of Saskatchewan’s hydro resources are limited and the possibility of import from Manitoba Hydro suffers from geographical distance. Federal government investments in transmission, buttressing exchange of power production from east to west, would address this deficiency. Further, government support for CCUS also could catapult CO_2_ into the status of an asset, prolonging its existence as a Saskatchewan actant.

This research has demonstrated strong synergy of NAS and the addition of actants and interessement from ANT deepening analysis and causal explanation. Actants combine natural resources present on the landscape together with actors and their narratives and illustrate sticky structural institutions and local contexts that could not have been explained otherwise. However, even ‘sticky’ institutions can change over time through the influence of distant action situations. Dynamics of polycentric governance are illustrated by the expansion of NAS to actants and interessement processes of change. Studying governance using NAS illustrates that its polycentric nature is neither top down nor collaborative. This study of NAS together with actants and interessement advances middle-range understanding of institutional change in complex systems of interconnecting systems and networks.

This research contributes to addressing the deficit of social science research in addressing complex problems of climate change and energy futures (Pellizzone et al. 2017; Sovacool et al. 2015) and pathways communities have for transitioning to sustainable energy futures (Devine-Wright and Wiersma 2020; Pidgeon et al. 2014). However, many questions remain. How do specific actors contribute to the narrative surrounding actants, what combinations of actors and actants effectively achieve net zero carbon futures, and what exact processes of interessement are required? Given that technology plays an important role, how do technology and actants inter-relate and how are narratives built in relation to techno-actants?

Further research exploring NAS and nodes, or actants, and their links through processes of interessement to create network assemblages in other geographies and sectors including water, food, and energy is required. This future research would determine if this research’s theoretical and methodological enhancement of NAS with ANT applies in different contexts and different geographical areas. Other methodologies in future research will also advance this enhanced study of NAS. A comprehensive empirical study of networks of actors that advance or oppose resources in the creation of nodes, or actants, might enhance understanding of links beyond the narrative analysis of this study. Future research focused on actor-networks, more specific assessment of flows (including information, finance, and ideas for new technology) in relation to links, or interessement, also might advance understanding of institutional change and causal mechanisms.

## Supplementary Information

Below is the link to the electronic supplementary material.

Supplementary file1 (DOC 409 kb)

## Footnotes

1 All are identified in the *Kyoto Protocol to the United Nations Framework Convention on Climate Change*.

2 In 2013, 24.21% of Saskatchewan’s Gross Domestic Product (GDP) came from mining, and oil and gas extraction (GS 2017a). Its contribution to the economy is recently illustrated. In 2015 Saskatchewan suffered a $4.8 billion decline in its nominal GDP, largely attributed to the decline in oil prices during 2015, which directly affected the value of exports and had indirect effects throughout the economy (GS 2017a). This resulted in losses of resource revenues of $1.3 billion, reduction in resource revenues of hundreds of millions, and resulted in government cutbacks in the 2017–2018 budget (GS 2017b).

3 Some Saskatchewan wind installations have been opposed because of impacts on animals in protected areas (CBC News 2015).
