# Supplementary material for: Dialectic narratives, hostile actors, and Earth’s resources in Saskatchewan, Canada
Source: Sustain Sci. 2022 Sep 20;18(1):285–301. doi: 10.1007/s11625-022-01214-y (PMC9485019; doi:10.1007/s11625-022-01214-y)

**Appendix Focus Group and Survey**

**Focus Groups**

For our data collection, we randomly contacted focus group participants in ten Saskatchewan cities using the published telephone numbers of Saskatchewan residents. Participants were advised of the purpose of the focus group (exploring Saskatchewan residents’ perceptions of energy futures), the risks and benefits of the research. Participants who agreed were invited to a focus group in their city. Between November 2019 and March 2020, 114 people attended ten (10) three hour focus group forums in ten cities. The cities included Regina, Saskatoon, Moose Jaw, Estevan, Weyburn, Yorkton, La Ronge, Prince Albert, Melfort and Swift Current.

Focus group transcripts were coded using the following codes:

Wind

Solar

Hydro

Coal

Natural Gas

Nuclear

SMRs

**Survey**

The survey comprises adult residents of Saskatchewan, randomly selected based on published Saskatchewan telephone numbers conducted in July and August 2020. In this phase 400 respondents were contacted and 136 (34%) agreed to fill in the survey.

The survey was comprised of Likert-scaled questions. The data was generated using answers to the survey question “Do you support or not support the use of each of the following energy sources to provide power in Saskatchewan? Please use a 10-point scale where 1 means you “don’t support at all” and 10 means you “completely support”. The energy sources include: Solar, Wind, Manitoba Hydro, SMRs, Nuclear Coal CCS and Natural Gas CCS.

Figure 4 describes respondents’ feedback on their support for different energy sources. The sample was a total of 136 response. The survey was analyzed through SPSS version 28, while diagnostic tests and ordered logistic regression were conducted using STATA 17.

In the analysis stage, the ranked responses were uploaded in SPSS to obtain descriptive statistics. Next, the responses were grouped such that responses from one (1) to five (5) were categorized as “Don’t support” and responses from 6 to 10 were categorised as “Support.” We further represented these responses as dummy variables using a binary code of 0 and 1, where 0 = Don’t support and 1= Support to obtain the error co-efficient of the distribution. Error coefficient can be described as the standard error of the mean of repeated estimates divided by the mean. It is a measure of how good an estimate is. See result below:


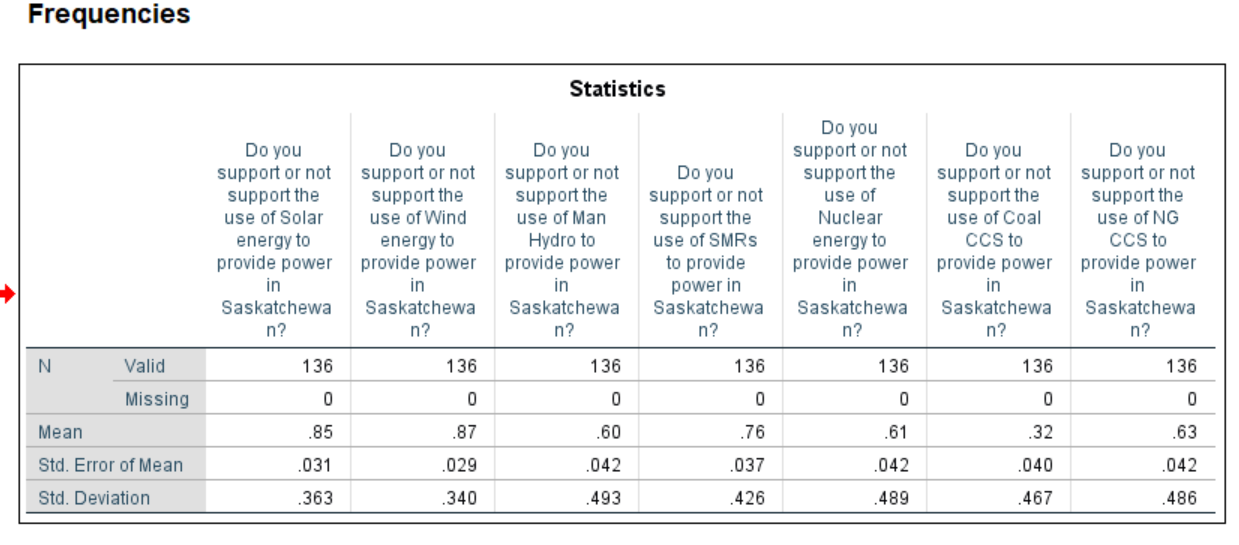


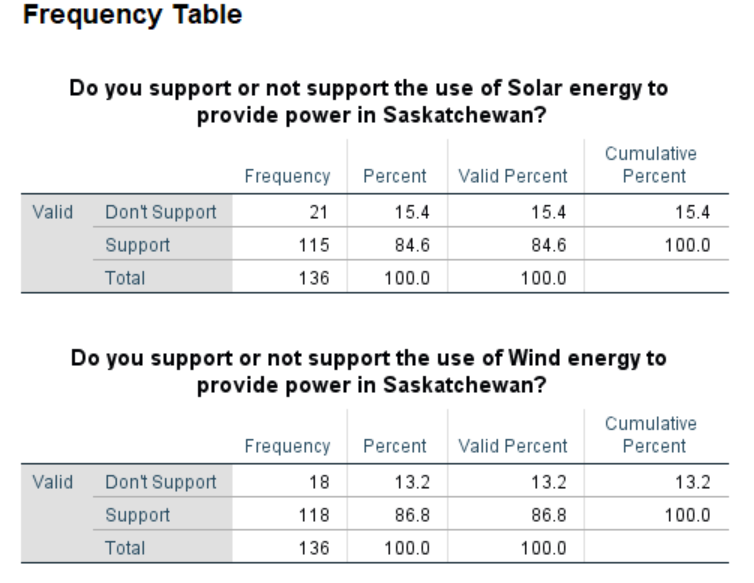


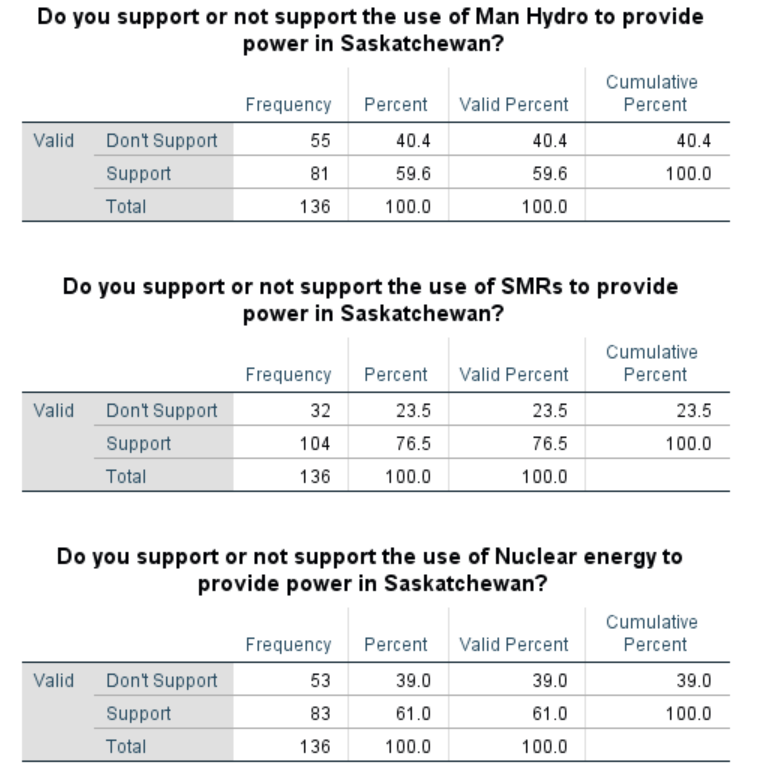


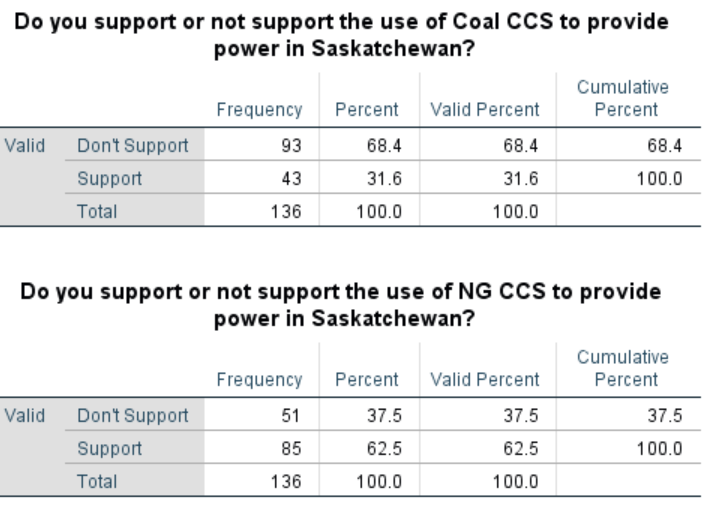

Supplement: Supplementary file 1 — Supplementary file1 (DOC 409 kb) [file 11625_2022_1214_MOESM1_ESM.doc]
